# The effect of image resolution of display types on accommodative microfluctuations

**DOI:** 10.1111/opo.12949

**Published:** 2022-02-01

**Authors:** Niall J Hynes, Matthew P Cufflin, Karen M Hampson, Edward AH Mallen

**Affiliations:** ^1^ Department of Optometry and Vision Sciences School of Applied Sciences University of Huddersfield Huddersfield UK; ^2^ School of Optometry and Vision Science University of Bradford Bradford UK; ^3^ Department of Engineering Science University of Oxford Oxford UK

**Keywords:** accommodative microfluctuations, digital eye strain, electronic displays, myopia

## Abstract

**Purpose:**

To determine whether accommodative microfluctuations (AMFs) are affected by the image resolution of the display type being observed. The effect of refractive error is also examined.

**Methods:**

Twenty participants, (10 myopes and 10 emmetropes) observed a target on four different displays: paper, smartphone, e‐reader and visual display unit screen (VDU), whilst their accommodative responses were measured using a continuous recording infrared autorefractor. The accommodative response and AMF measures comprising low frequency components (LFC), high frequency components (HFC) and the root mean square (RMS) of the AMFs were analysed.

**Results:**

A significant increase in LFC power was observed for the paper stimulus when compared to the VDU and smartphone conditions. Myopes demonstrated a significantly higher LFC and mean accommodative response compared to emmetropes across the four displays. A significant difference in the mean AR between the displays with the lowest and highest resolution was found. A higher mean AR was found with higher resolution of the image. The HFC and RMS accommodation were not affected by display type.

**Conclusion:**

The mean accommodative response and the mean LFC power appear to respond differently depending on the type of display in use. Higher resolution devices showed a reduced lag of accommodation to the accommodative demand; however, this may cause a lead of accommodation in myopes for higher resolution display types.

**Key points**

- Displays with a higher resolution appear to decrease the lag of accommodation and increase the mean power of the low frequency component in accommodative microfluctuations.
- Refractive error appears to modify the effect of image resolution, with myopes having a decreased lag of accommodation relative to emmetropes.
- It may be necessary to consider that non‐symptomatic image resolutions range between an excessive lag of accommodation in lower resolutions and a lead of accommodation with higher resolutions.

## INTRODUCTION

Digital eyestrain (DES), also known as computer vision syndrome, is a term used to describe where an individual has ocular and visual symptoms due to prolonged use of a range of digital display devices including computers, smartphones and tablets.^1^, ^2^ Visual symptoms include headaches, eyestrain, ocular discomfort, dry eye, diplopia and blurred vision either during or after prolonged visual display unit (VDU) use.^1^, ^2^, ^3^, ^4^ Numerous factors are thought to cause DES including uncorrected refractive error,^3^, ^5^ time spent working on VDUs,^3^, ^6^ working distance,^3^, ^7^ presbyopia,^3^, ^8^ oculomotor responses^3^, ^7^ and dry eye. Originally these symptoms corresponded to individuals who spent prolonged amounts of time working on VDUs. However, given the increase in the range of display devices available at the present time, including smartphones and electronic reading devices, the condition has become more prevalent^4^ with 80% of American adults reporting using a digital device for at least 2 hours a day and 67% using two digital devices simultaneously.^9^ A recent study in India showed that 20% of 11‐year‐olds used digital devices, rising to 50% of 17‐year‐olds.^10^ Studies have suggested that there is an increased lag of accommodation following smartphone use, surpassing that of printed text.^11^, ^12^

Early research examining eye strain, the accommodative response (AR) and accommodative microfluctuations (AMFs) found that the power of the low frequency component (LFC) was significantly increased following an hour of computer work.^13^ The same finding was not observed following an hour of paper‐based work, with no significant difference between pre‐ and post‐work levels. It has been suggested that AMF analysis could have the potential to be useful in determining more subtle changes in symptomatic individuals suffering from DES. However, the nature of the requirements for assessing AMFs make this difficult, with much of the research conducted on specifically modified autorefractors in research labs rather than commercially available, purpose‐built equipment.^4^, ^14^, ^15^, ^16^

It is important to consider refractive error in this experiment, given the numerous studies that point towards a positive correlation between myopia and near work.^17^, ^18^, ^19^ Recent cross‐sectional studies have suggested that use of digital and paper‐based display types is associated with the development of myopia.^20^, ^21^ The increase in education being delivered digitally rather than paper‐based has been suggested as an area requiring further investigation in terms of the development of myopia.^22^ Further, myopes have been found to have larger AMFs than emmetropes.^23^, ^24^, ^25^ It has been suggested that the increased variability for myopic participants may be due to reduced blur sensitivity caused by an increased depth of focus.^24^

Liquid crystal display (LCD) and e‐ink display devices, along with paper, have become popular for reading.^26^, ^27^ There are several factors that may be considered when comparing and contrasting display types, including image resolution, luminance and refresh rate. A major difference between LCD and e‐ink displays is that e‐ink displays do not need to refresh, therefore eliminating any flicker.^26^ Image resolution and luminance vary between display devices and can be adjustable.

The purpose of this experiment is to determine whether AMFs are affected by the image resolution of a variety of commonly used display types. As technology develops, it is important to note any changes in their effect on the accommodation system compared to earlier display types. The effects of refractive error are also examined.

## METHODS

### Participants

Twenty participants (10 myopes and 10 emmetropes) took part in the study (details in Table 1). Classification was based on mean spherical equivalent refractive error, which was <−0.50 D for myopes and –0.25 D to +0.75 D for emmetropes.^28^, ^29^ Participants with astigmatism greater than 1.00 D were excluded. Monocular and binocular vision or visual acuity (VA) was measured using a Bailey‐Lovie logMAR chart, with all participants achieving +0.02 logMAR or better in their right eye, and all were free of any ocular pathology.

**TABLE 1:** Information regarding participants in the experiment

Refractive group	Emmetropes	Myopes
No. of participants	10	10
Mean age (years)	23.13 ± 2.88	23.98 ± 4.61
Range (years)	20–29	20–33
Mean refractive error (D)	+0.26 ± 0.25	−4.67 ± 1.93
Range of mean Sph (D)	−0.12 to +0.75	−2.50 to −7.37
Range of cylinder (D)	0.00 to −0.50	0.00 to −1.00

Note

± values refer to one standard deviation of the mean.

Abbreviation: D, dioptre.

Soft contact lenses were used to correct the myopes. Previous research has shown that these lenses do not have an effect on AMFs.^30^ In most cases the participants own habitual contact lens correction was used; however in the case of non‐contact lens wearers, they were fitted with daily disposable lenses (1‐Day Acuvue Moist, Johnson & Johnson, jnjvisionpro.com) and were allowed approximately 20 min to adapt to the lenses. Any residual refractive error after contact lenses correction was less than 0.50 D mean spherical error as measured using the Shin‐Nippon SRW‐5000 autorefractor (shin‐nippon.jp).

The amplitude of accommodation was measured using an RAF rule^31^ to ensure that participants had a minimum amplitude of accommodation of 6.00 D, i.e., double the accommodative demand required for the experiment. The study was approved by the Biomedical, Natural and Physical Sciences Research Ethics Panel at the University of Bradford and this study was undertaken in accordance with the Declaration of Helsinki.

### Instrumentation

The accommodative response was measured using a Shin‐Nippon SRW‐5000 autorefractor modified for continuous recording whilst allowing binocular open‐field viewing of targets.^32^, ^33^ This has been used in similar prior experiments.^14^, ^15^, ^23^ A forehead and chin rest were used to secure the head of participants to reduce any ocular movement. The Shin‐Nippon SRW‐5000 autorefractor requires a pupil size greater than 2.9 mm, so only participants with pupil sizes exceeding this value during near fixation were recruited.

Four different display types were used to present an identical high contrast black Maltese cross target on a white background. These were white paper, a 17” LCD monitor (MultiSync LCD175VXM+, NEC, nec‐display.com), a smartphone (iPhone SE, Apple, apple.com) and an e‐reader (Kindle Paperwhite, Amazon, amazon.com).

### Tasks

The tasks were completed by participants in a laboratory illuminated by light‐emitting diode (LED) tube lighting measuring 880 lux.

**FIGURE 1:**
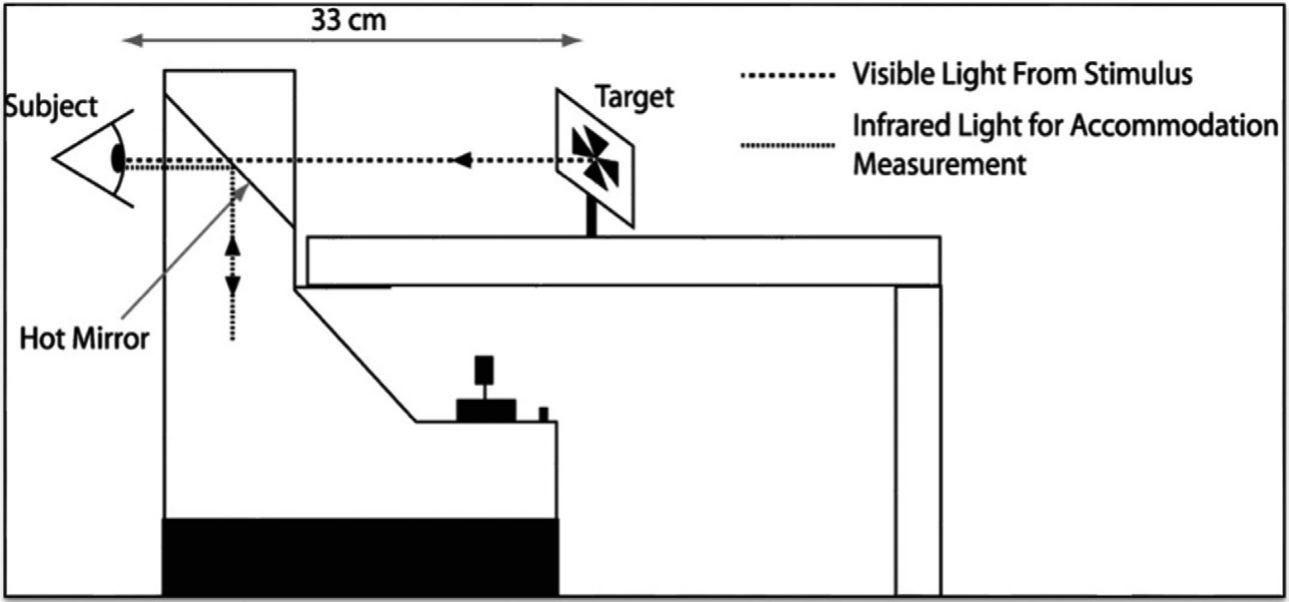
Participants observed the target from a distance of 33 cm whilst viewing binocularly through a Shin‐Nippon SRW‐5000 autorefractor

Participants were required to observe a high contrast Maltese cross target at 33 cm on four different types of displays (Figure 1). They were instructed to observe the target and keep it as clear as possible at all times while keeping their head as still as possible and blinking normally. The fixation target subtended a visual angle of 0.52 degrees for all four conditions. Luminance settings for the smartphone, e‐reader and visual display unit (VDU) screen were adjusted to match the paper target. The luminance for the display types was 61.1, 61.4, 60.9 and 61.3 cd/m^2^ for the paper, smartphone, e‐reader, and VDU screen, respectively.

The screen sizes for the e‐book and smartphone measured 15.24 cm and 10.16 cm diagonally, respectively. Due to reflections from the peripheral areas of the VDU screen, masking was introduced to prevent degradation of the accommodation measurements taken by the autorefractor.

Resolution varied amongst the displays. The paper target was printed using a laser printer (PageWide Managed Color MFP E58650, Hewlett‐Packard, hp.com) printed at 600 dots per inch (dpi). The smartphone, e‐reader and computer monitor had resolutions of 326, 300 and 96 dpi, respectively.

The accommodative response was measured for approximately 20 s for each display type. A baseline distance reading was taken first, followed by the four experimental conditions presented in a random order. This was repeated three times for each participant, restarting with the distance baseline measurement each time.

### Analysis

Three accommodation traces, lasting 20 s in duration, were recorded for each of the four tasks. When analysing the mean accommodative response, blinks were removed from the data in an Excel program (Microsoft, microsoft.com) by identifying accommodative response measures that were ≥2 D away from the accommodative demand, and then replaced with an average of the five previous data points before the blink.^34^ The three measures for each task were averaged.

The root mean square of the AMFs (RMS accommodation) and the powers of the LFC and HFC were calculated using MATLAB (MATLAB R2013a, MathWorks,mathworks.com). Blinks were removed from the data by identifying accommodative response measures that were 2 D away from the accommodative demand and replacing them using interpolation, as per Hampson and colleagues.^15^, ^35^ The LFC and HFC were calculated using fast Fourier transforms (FFTs). The three measures for each task were averaged. The LFC was defined as frequencies between 0 and 0.6 Hz and the HFC between 1.0 and 2.1 Hz.^25^, ^36^ The RMS accommodation measures were included as an indicator of overall variability for the AMFs and to be comparable with previous studies.^25^

Data analysis was carried out using repeated measure analyses of variance (ANOVAs) through SPSS version 22.0 (IBM, ibm.com) to compare the mean AR, LFC, HFC and RMS of the AMFs across the four display types. Results were deemed statistically significant when *p*‐values of <0.05 were present.

## RESULTS

The results for the mean AR, mean powers of the LFC and HFC and RMS accommodation are shown in Table 2.

**TABLE 2:** Mean values and standard deviation (SD) for the pooled data, emmetropes and myopes for the mean accommodative response, low frequency components (LFC), high frequency components (HFC), RMS and root mean square (RMS) for the display types: paper, phone, e‐reader and visual display unit (VDU)

Display	Paper	Phone	E‐reader	VDU	*p*‐Value
Pooled data					
LFC (D^2^/Hz)	8.23 ± 3.78	7.67 ± 3.61	7.20 ± 3.43	5.95 ± 2.99	<0.001*
HFC (D^2^/Hz)	0.01 ± 0.01	0.01 ± 0.00	0.01 ± 0.01	0.01 ± 0.01	0.15
RMS (D)	0.31 ± 0.07	0.30 ± 0.06	0.32 ± 0.08	0.30 ± 0.06	0.45
Mean AR (D)	2.79 ± 0.68	2.65 ± 0.65	2.59 ± 0.64	2.36 ± 0.57	<0.001*
Emmetropes					
LFC (D^2^/Hz)	5.98 ± 2.04	5.47 ± 1.83	5.43 ± 2.31	4.43 ± 1.34	0.06
HFC (D^2^/Hz)	0.01 ± 0.01	0.01 ± 0.01	0.01 ± 0.01	0.01 ± 0.01	0.16
RMS (D)	0.31 ± 0.08	0.29 ± 0.07	0.34 ± 0.11	0.29 ± 0.07	0.21
Mean AR (D)	2.40 ± 0.43	2.28 ± 0.41	2.23 ± 0.48	2.07 ± 0.32	0.05*
Myopes					
LFC (D^2^/Hz)	10.47 ± 3.84	9.87 ± 3.67	8.96 ± 3.53	7.47 ± 3.45	0.005*
HFC (D^2^/Hz)	0.01 ± 0.00	0.01 ± 0.00	0.01 ± 0.01	0.01 ± 0.01	0.70
RMS (D)	0.31 ± 0.06	0.32 ± 0.06	0.30 ± 0.04	0.30 ± 0.04	0.85
Mean AR (D)	3.18 ± 0.66	3.03 ± 0.66	2.95 ± 0.58	2.66 ± 0.63	0.01*

Note

± values refer to 1 standard deviation of the mean. *p*‐Values for the effect of display type in the four groups are included in the last column. Significant effects are denoted by use of an asterisk.

Abbreviation: AR, accommodative response.

### Mean accommodative response

**FIGURE 2:**
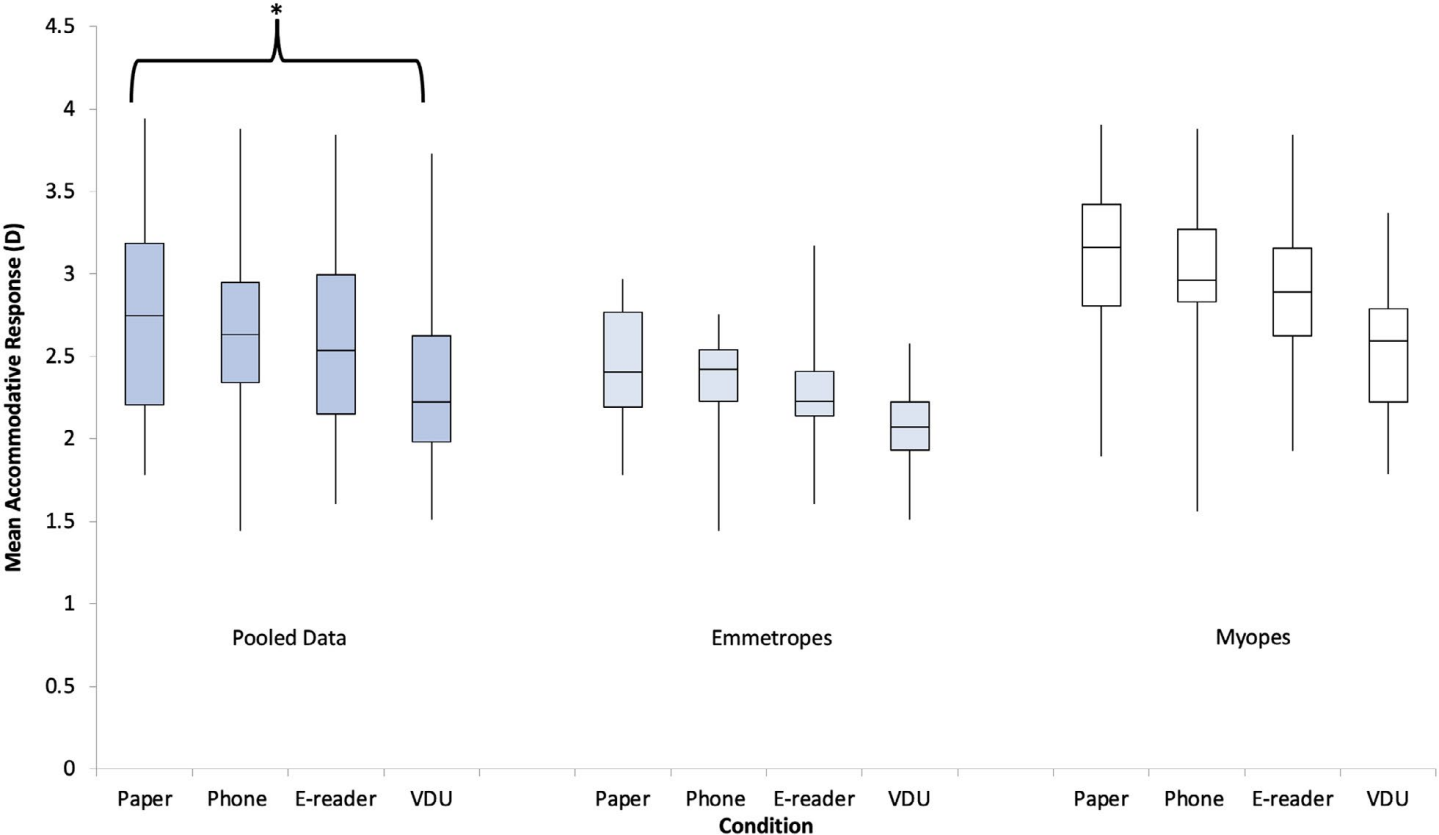
Box plots demonstrating the mean accommodative response for pooled data, emmetropes and myopes for the display types: paper, e‐reader, phone and visual display unit (VDU). A repeated measures analysis of variance (ANOVA) with Bonferroni post‐hoc analysis found a significant main effect depending on the display typed used. Pairwise analyses demonstrated a significant difference in the mean accommodative response (AR) between the paper and VDU displays for the pooled data. This is denoted by an asterisk in the box plot. A repeated measures ANOVA with Bonferroni post‐hoc analysis found a significant difference between the refractive groups

A significant main effect of display type was found across participants (*F*_3,54_ = 7.60, *p* < 0.001), following a repeated measures ANOVA. Pairwise comparisons were analysed with a significant effect demonstrated between the paper and VDU conditions only (*p* < 0.01) (Figure 2).

A significant effect between the myopic and emmetropic refractive groups was found (*F*_1,18_ = 11.31, *p* < 0.005) for the overall mean accommodative responses, with a higher response noted in myopes. A significant main effect was found for display types for both myopes (*F*_3,27_ = 4.64, *p* = 0.01) and emmetropes (*F*_3,27_ = 2.98, *p* < 0.05) when the groups were examined separately. No significant differences were found in pairwise analyses for myopes and emmetropes. No significant interaction was found between the refractive error and the display type (*F*_3,54_ = 0.40, *p* = 0.75).

**FIGURE 3:**
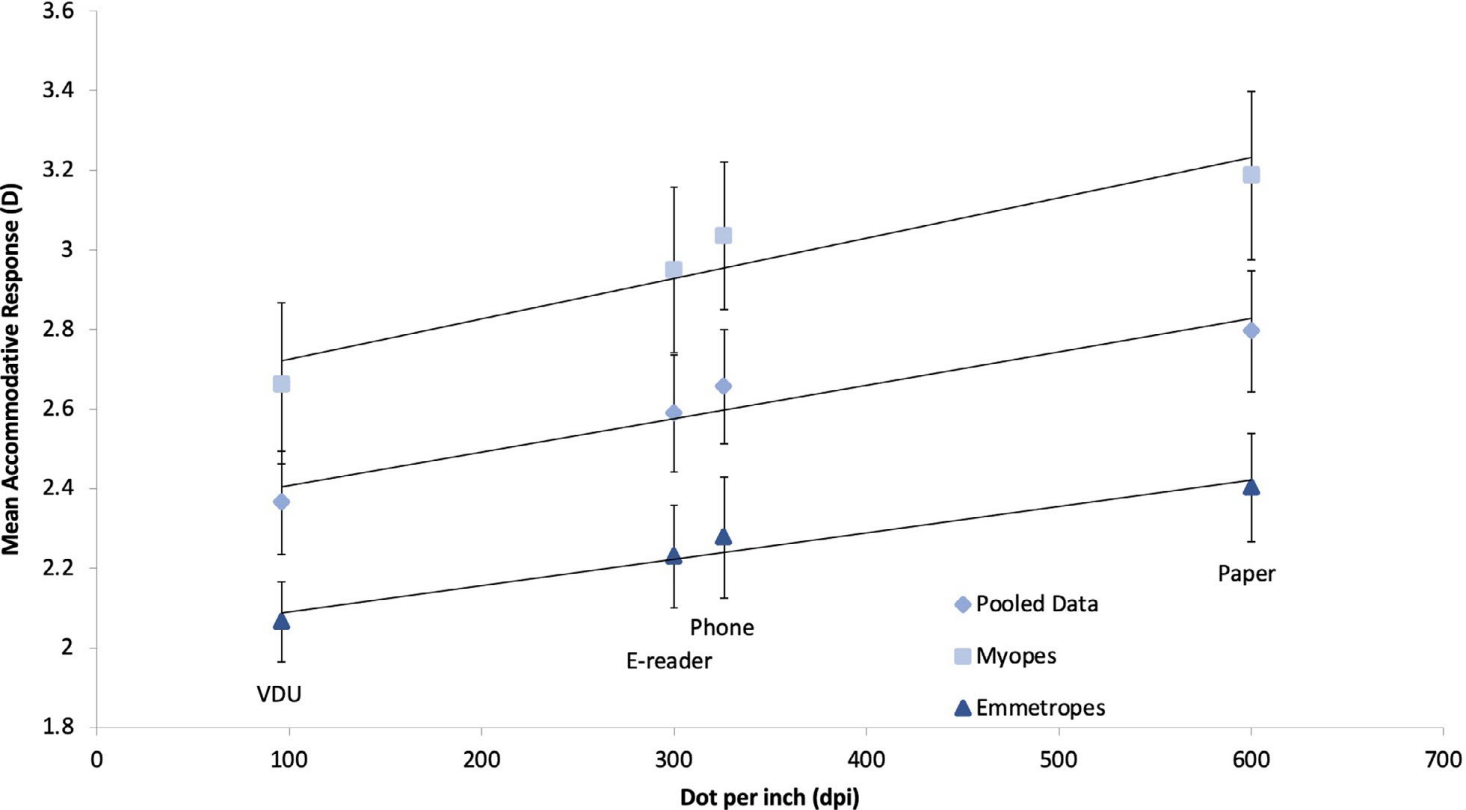
Scatter graph for the mean accommodative response against the image resolution of the display types: paper (600 dpi), phone (326 dpi), e‐reader (300 dpi) and visual display unit (VDU) (96 dpi) for myopes, emmetropes and pooled data. A significant correlation between the mean accommodative response and the image resolution of the display types was found for all four groups (all *p* values < 0.05) Error bars are standard errors of the mean

There was a strong positive correlation between the mean accommodative response and the image resolution of the display types for the pooled data, *r* = 0.97, 95% BCa CI [0.95, 1], *p* = 0.03. There was also a strong positive correlation separately for myopes, *r* = 0.96, 95% BCa CI [0.94, 1], *p* = 0.04 and emmetropes, *r* = 0.98, 95% BCa CI [0.97, 1], *p* = 0.02 (Figure 3). This indicates that increasing the stimulus dpi results in an increase in the accommodation response.

### Root mean square accommodation

**FIGURE 4:**
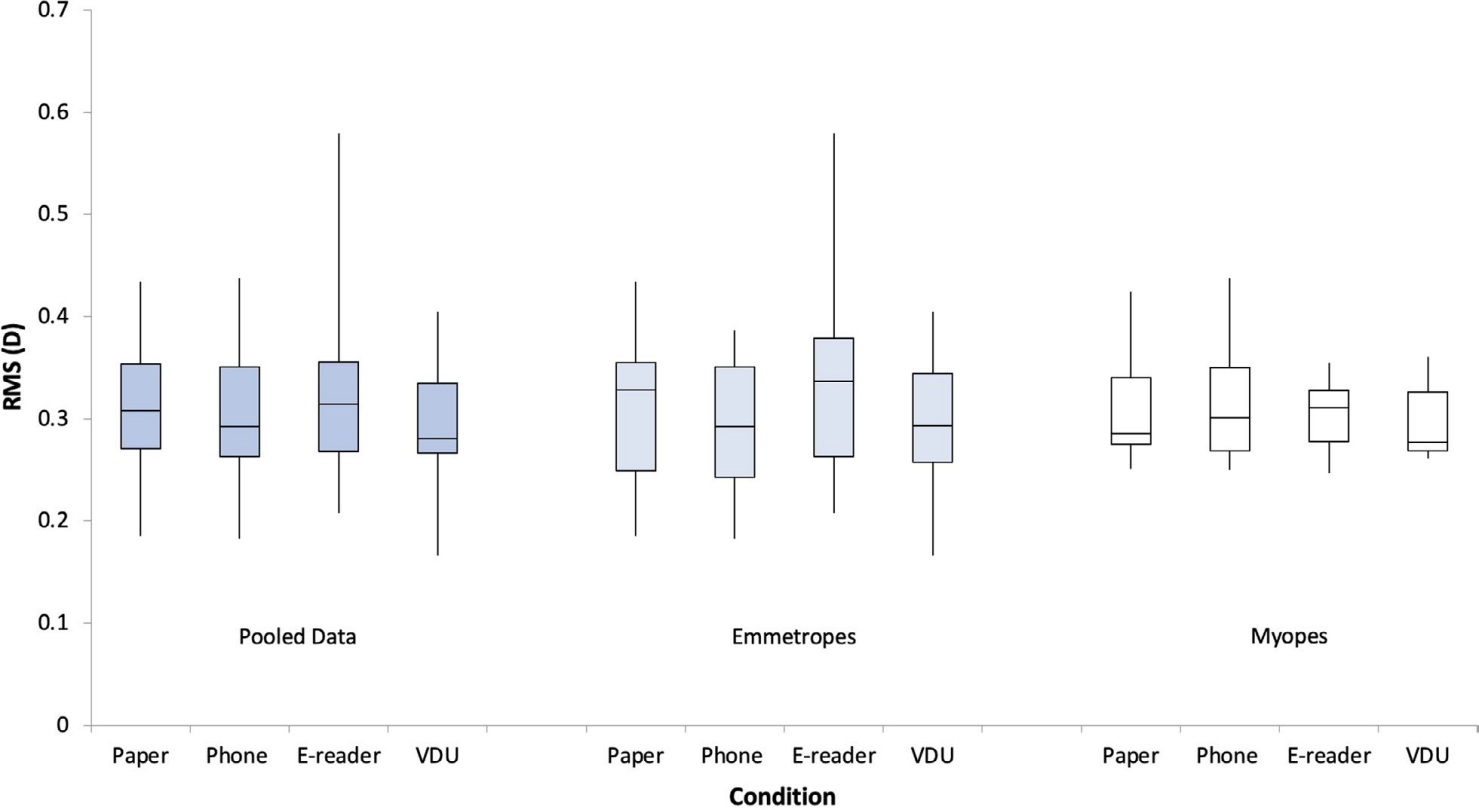
Box plots demonstrating the root mean square (RMS) accommodation for the pooled data, emmetropes and myopes for the display types: paper, phone, and e‐reader and visual display unit (VDU). Repeated measures analyses of variance (ANOVAs) with Bonferroni post‐hoc analysis found no significant differences across the three conditions

There was no significant effect of refractive error group for RMS accommodation (*F*_1,18_ = 0.001, *p* = 0.97), so data were pooled into a single group. RMS accommodation was also found to be unaffected by display type (*F*_3,54_ = 0.90, *p* = 0.45) (Figure 4).

### Power spectrum analysis

**FIGURE 5:**
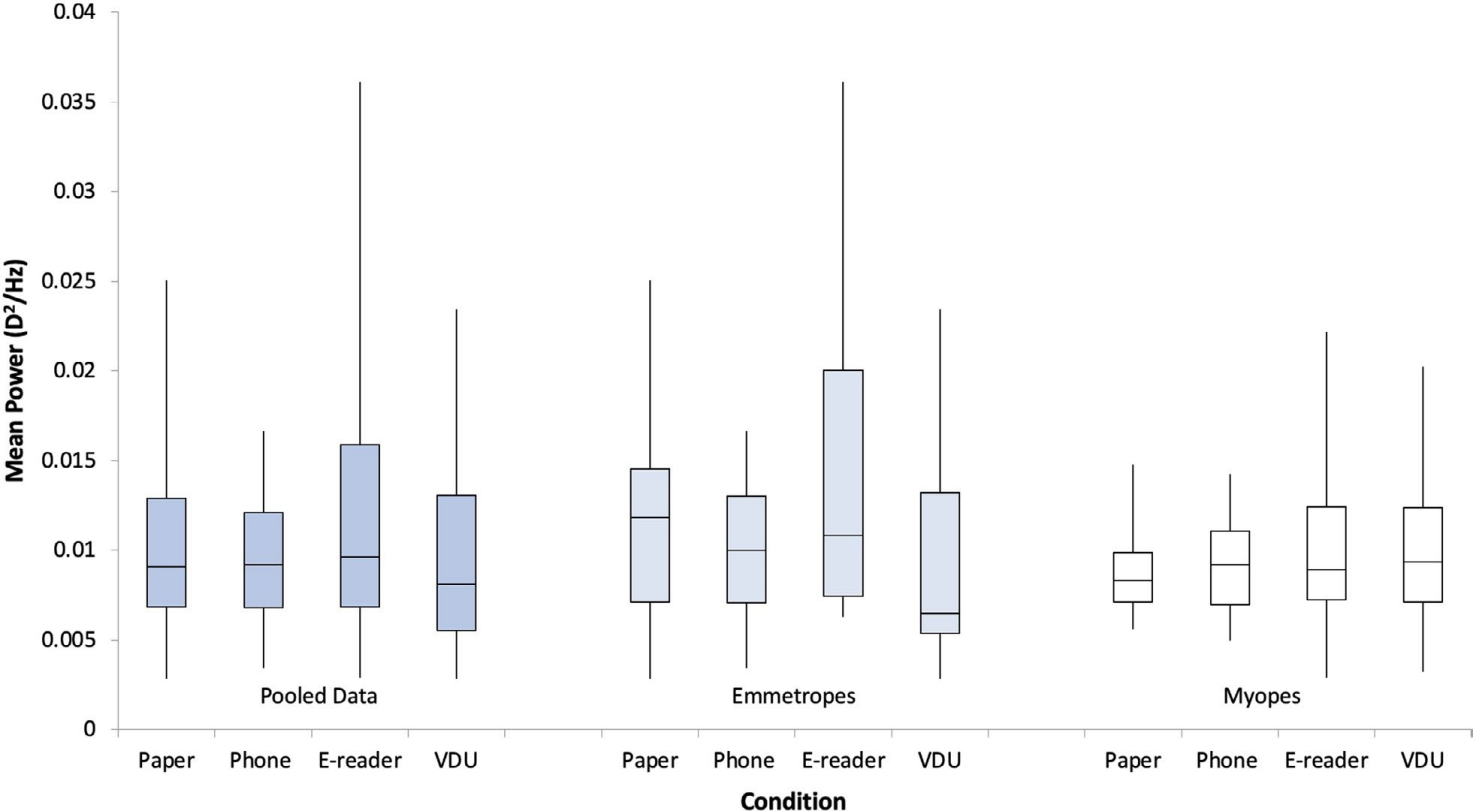
Box plots demonstrating the high frequency component (HFC) for the pooled data, emmetropes and myopes for the display types: paper, phone, e‐reader and visual display unit (VDU). Repeated measures analyses of variance (ANOVAs) with Bonferroni post‐hoc analysis found no significant differences across the three conditions

The accommodative traces underwent FFT to isolate the LFC and HFC of the signal. The effect of the display type on each of these components was examined. There were no significant differences found for refractive error types for the HFC (*F*_1,18_ = 0.87, *p* = 0.36), therefore data were pooled. A repeated measures ANOVA found no main significant effect for display types in the HFC (*F*_3,54_ = 1.95, *p* = 0.15) (Figure 5).

**FIGURE 6:**
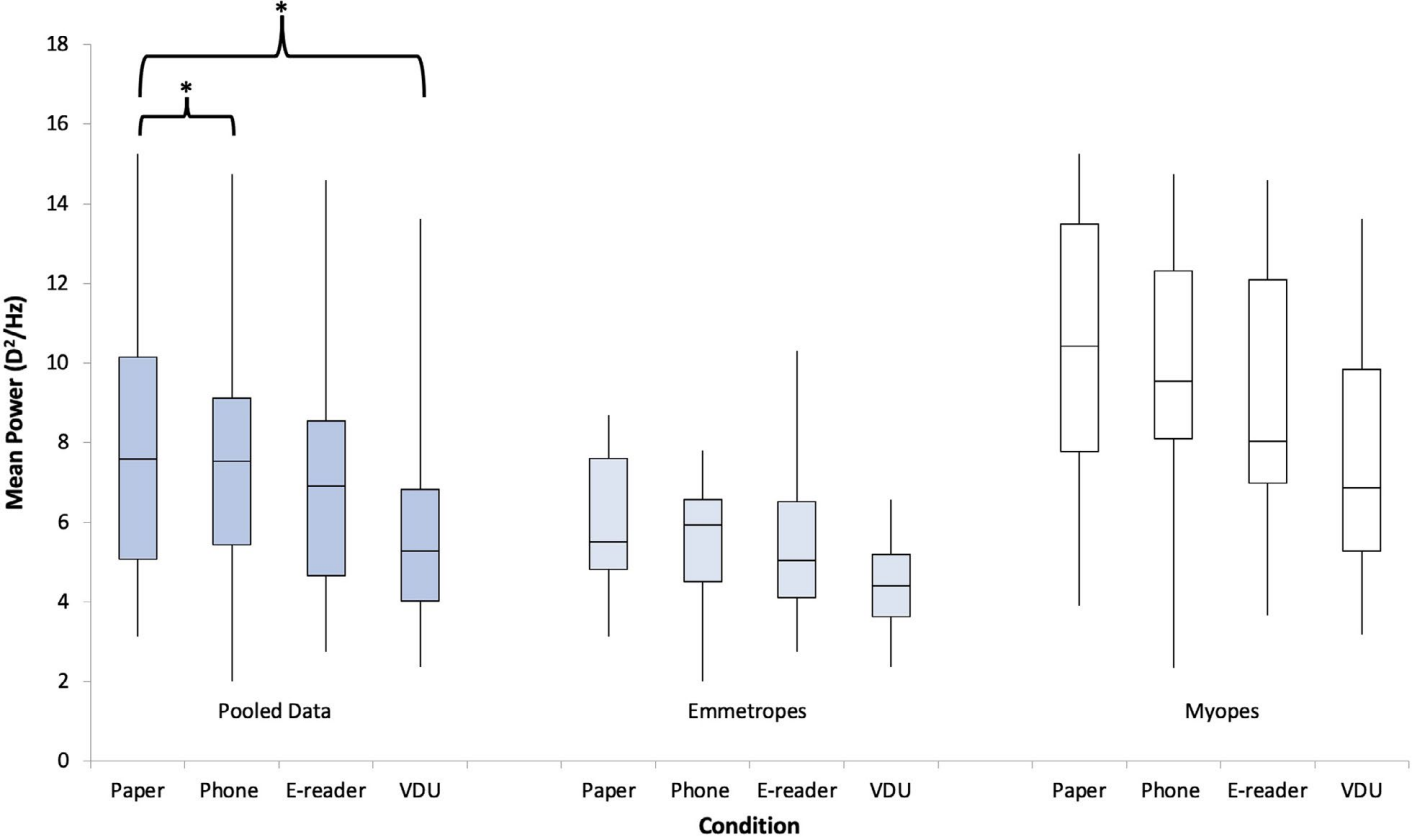
Box plots demonstrating the low frequency component (LFC) for the pooled data, emmetropes and myopes for the display types: paper, phone, e‐reader and visual display unit (VDU). A repeated measures analysis of variance (ANOVA) with Bonferroni post‐hoc analysis found a significant main effect depending on the display type used for the pooled data. Pairwise analyses revealed a significant difference in the mean accommodative response (AR) between the paper and VDU displays and the paper and smartphone displays. These are denoted by an asterisk in the box plot. A repeated measures ANOVA with Bonferroni post‐hoc analysis found a significant difference between the refractive groups

A significant effect between the myopic and emmetropic refractive groups was found (*F*_1,18_ = 11.31, *p* < 0.005) in the LFC (Figure 6). There was no interaction found between the display types and the refractive error (*F*_3,54_ = 1.02, *p* = 0.39). A significant main effect was found for display type in myopes (*F*_3,27_ = 5.27, *p* = 0.005), but not for emmetropes (*F*_3,27_ = 2.72, *p* = 0.06) separately. There were no significant differences found between the different display types for myopes and emmetropes separately when pairwise analyses were examined, therefore, data were pooled.

A main significant effect was found across participants depending on the display type used (*F*_3,54_ = 7.87, *p* < 0.001), following a repeated measures ANOVA. Pairwise comparisons were analysed with a significant effect demonstrated between the paper and VDU conditions (*p* < 0.01) and paper and smartphone conditions (*p* < 0.05). There were no more significant differences noted between the rest of the display types combinations for pairwise comparisons.

**FIGURE 7:**
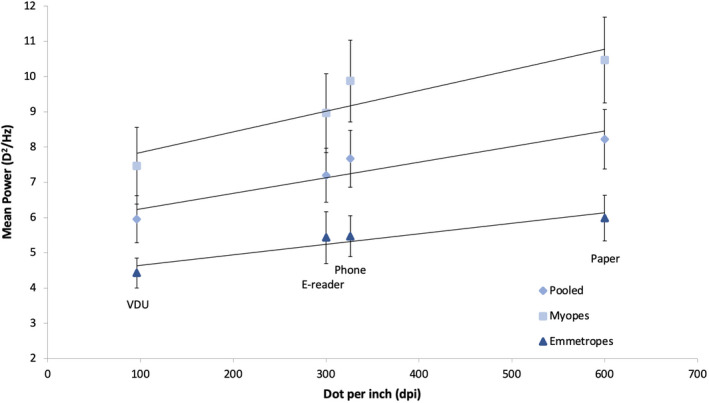
Scatter graph demonstrating for the mean power of the low frequency component (LFC) against the image resolution of the display types: paper (600 dpi), phone (326 dpi), e‐reader (300 dpi) and visual display unit (VDU) (96 dpi) for myopes, emmetropes and pooled data. No significant correlation between the mean power of the LFC and the image resolution of the display types was found for the pooled data, myopic or emmetropic groups (*p* = 0.06, *p* = 0.07 and *p* = 0.05, respectively). Error bars are standard errors of the mean

There was no significant correlation found between the LFC and the image resolution of the display types for the pooled data, *r* = 0.94, 95% BCa CI [0.89, 1], *p* = 0.06, for myopes, *r* = 0.93, 95% BCa CI [0.78, 1], *p* = 0.07 or emmetropes, *r* = 0.95, 95% BCa CI [0.94, 1], *p* = 0.05 (Figure 7).

## DISCUSSION

A significant increase in the mean power of the LFC was observed for the paper condition when compared to the VDU and smartphone conditions. Myopes demonstrated a significantly higher mean power of the LFC and mean AR compared with emmetropes across the four displays. A strong positive correlation was noted for myopes and emmetropes between the mean accommodative response and the image resolution of the display types. The HFC and RMS accommodation were found not to be affected by display type.

### Power frequencies of accommodative microfluctuations (AMFs) and display type

Previous studies have suggested that display type may influence AMFs.^4^, ^13^, ^37^, ^38^ Iwasaki and Kurimoto^13^ found an increase in the LFC when participants used a computer compared to a paper target. This differs from the findings of our study, where the mean power of the LFC was highest for the paper display and lowest for the VDU display. In Iwasaki and Kurimoto's study this was only apparent once the participants had completed an hour‐long task searching for names on the display they were using. The LFC was defined as between 0 and 1.5 Hz, whereas this study defined the LFC as between 0 and 0.6 Hz, similar to previous investigations.^25^, ^39^, ^40^, ^41^ The value used by Iwasaki and Kurimoto overlaps into the HFC range used for our experiment (1.0–2.1 Hz), making it difficult to directly compare results. The accommodative response was recorded at optical infinity in low luminance conditions (3–5 lux) leading to no accommodative demand and lower luminance compared to the present study. Given the effect that accommodative demand^23^, ^42^, ^43^ and luminance^14^, ^40^ have on AMFs, it is possible that this is a contributing factor to the differences found between the two experiments. Any possible effect of image resolution was not mentioned, which may have also resulted in a difference in the LFC for the displays used as found here, where accommodative demand and luminance levels were kept constant across the display types.

Gray and colleagues^37^ examined AMFs and pupil size during sustained viewing of several different displays. Five participants were presented with a task identifying typographical errors on five different displays. No differences were found in the accommodative response; however, one participant exhibited an increased LFC for paper and an electroluminescent panel after undertaking the task for 20 min. Target contrast and angular subtense were varying factors depending on the display used in that study,^36^ whereas these parameters remained constant throughout this investigation. A lower accommodative demand of 2.00 D was used in their experiment, which as with Iwasaki and Kurimoto, may be a factor in the results when compared to the 3.00 D accommodative demand of our study. However, the observed increase in the LFC is similar to the results presented here. A different LFC range (0.3–0.6 Hz) was used by Gray and colleagues.^37^ The narrower frequency band is another possible reason for the more apparent differences in the LFC between display types that was found in this experiment.

It has been proposed that the measurement of the HFC during the use of various display types can be a method of measuring visual stress.^44^ Jeng and colleagues^44^ concluded that the visual system was more strained when observing 3D videos compared to 2D videos, and when viewing material on a LCD TV (55 dpi) compared to a laser projector (29 dpi) at 2 m. In our experiment we found no significant difference between the HFC. One potentially important difference between the two studies is that we took all our readings at the same visit, whereas readings were taken on separate days for each condition in the other. As the HFC is correlated with the arterial pulse,^45^ it is possible that varying arterial frequencies may have an effect; an effect that is unlikely to impact the results of our experiment where participants were completing similar tasks back‐to‐back in a sedentary position.

In their study, Hue and colleagues^38^ conducted two separate experiments where they compared the AR between a first‐generation iPod touch (163 ppi) and paper in one cohort of 20 young participants, and the AR between a Kindle eBook reader (167 ppi) and paper in a different group of 20 young participants. An increased lag of accommodation was found in the iPod touch group compared to paper, but not in the Kindle group. The image resolution was similar between the two digital devices; however, they were not compared to one another. Given that there were two separate cohorts involved, this may explain why there was a difference in the AR in one group and not the other. Screen luminance was not reported by Hue and colleagues which also may have had an effect. The difference in screen sizes, 3.5 inches for the iPod touch and 6 inches for the Kindle, could be another potential cause for the effect. However, in our experiment there was no significant difference between the e‐reader and phone displays which had similar luminances and image resolutions but different screen sizes. A significant difference was found in the mean power of the LFC between the paper and VDU displays, which had the same screen size. A significant difference in the mean power of the LFC was also found between the paper and the phone displays, which had different display sizes. Given the correlation seen in our experiment between image resolution and the mean power of the LFC, along with the mean AR, it is more likely that image resolution, rather than screen size, has an effect on these measures.

### Refractive error

In the present study we found that the mean power of the LFC of the AMFs was greater in myopes than in emmetropes. This is similar to results reported in various other studies investigating the role of AMFs.^14^, ^23^, ^24^, ^25^, ^46^, ^47^ Our experiment also showed no interaction effect on the mean power of the LFC or HFC between refractive error and display type. Day and colleagues^23^ investigated the effect that changing the accommodative demand had on AMFs. They found that refractive error and accommodative demand had a significant effect on LFC power, with larger values seen in myopes compared with emmetropes. The lack of interaction between refractive error and accommodative demand found in their experiment, and refractive errors and image resolutions of display types found in our study, would suggest that refractive error has an independent effect on the LFC. Based on this, it is doubtful that the image resolution of the display types used here had an effect on the differences found between myopes and emmetropes, with myopes appearing to be generally predisposed to having an increased mean power of the LFC relative to that of emmetropes.

An increased depth of focus in myopes has been suggested as a possible reason for the higher AMFs noted in myopes compared with emmetropes.^24^, ^48^ Harb and colleagues^21^ hypothesised that the increased depth of focus reduced blur sensitivity, which led to an increase in accommodative variability while maintaining clear vision. In their study, emmetropes and myopes had approximately the same mean accommodative response; however myopes demonstrated greater accommodative variability suggesting that they may have the ability to tolerate greater AMFs. This varies with the results of our study where myopes exhibited a larger mean AR than emmetropes. The myopic response varied more for the mean AR, albeit for some displays more than others, and the mean power of the LFC relative to emmetropes which may be explained by the greater error allowed by an increased depth of focus. This finding contrasts with the generally accepted finding that myopes tend to have a larger lag of accommodation than emmetropes.^47^, ^49^ However, it has been shown that corrected adult myopes can have a smaller lag of accommodation than emmetropes.^50^ Nakatsuka and colleagues investigated the lag of accommodation using a similar binocular open‐field infra‐red autorefractor, finding that the lag of accommodation was smaller for myopes than emmetropes, with it being further reduced under binocular versus monocular conditions at a range of accommodative demands. A similar study conducted by the same researchers on children found the lag of accommodation was greater in myopes relative to emmetropes.^49^ This may explain our finding where the corrected adult myopes had a reduced lag of accommodation compared with emmetropes. However, this does not explain the lead of accommodation found for the display types with higher image resolutions which requires further investigation.

### Possible relationship between image resolution and accommodation

A potential reason for the effect of display type on the mean AR and the mean LFC power in AMFs could be related to the image resolution of the display. There was a significant positive correlation between the mean accommodative response and the resolution of the displays used in the experiment. This suggests that a reduced lag of accommodation occurs when viewing higher resolution displays, whereas a larger lag of accommodation may occur for lower resolution displays. This appears to be more obvious in emmetropes who had a larger lag of accommodation relative to myopes, whereas myopes had a lead of accommodation for higher image resolutions. Previous research assessing the mean accommodative response with near visual tasks on different handheld display types did not find a significant difference.^51^ Moulakaki and colleagues compared the accommodative response to a Maltese cross target at accommodative demands of 1.00 D, 2.00 D, 3.00 D and 4.00 D following 10 min silently reading text on an IPad mini (162 dpi) or iPhone 4S (330 dpi) in 18 participants. They found no significant difference in the accommodative response between the two devices across these accommodative demands. One possible reason could be related to the difference in image resolution between the two display types of 168 dpi. In this study, we found no significant difference between the paper display and either the smartphone (274 dpi) or the e‐book (300 dpi). However, there was a significant difference between paper and the VDU (504 dpi). Therefore, it could be hypothesised that a difference of greater than 300 dpi is required to achieve a change in the accommodative response when image resolution is isolated as a factor. Another difference between the two experiments is that the accommodative response was measured whilst participants were observing the display type, whereas all participants in Moulakaki and colleagues^51^ experiment observed the same target following a period of time reading from the test display types.

A positive trend was noted for both myopes and emmetropes between the power of the LFC and the image resolution of the display types; however this proved to be not significant. A larger range of image resolution may have shown a significant effect on the power of the LFC, with the trend appearing to be similar to that of the mean accommodative response.

### Image resolution and digital eye strain

Previous research has recommended that higher image resolutions should be used in digital displays.^52^, ^53^, ^54^ Ergonomically, Ziefle^52^ found that task performance was better with higher resolution displays relative to lower resolution displays. Participants completed tasks on two different cathode‐ray tube (CRT) displays and a paper display with image resolutions of 60, 120 and 255 dpi, respectively. Reading accuracy and speed were found be significantly higher on the paper display, with an overall trend showing an increase in these factors in line with an increase in the image resolution. This is similar to our study where a significant difference was found in the AR and the mean power of the LFC between the highest and lowest image resolutions displays. The image resolution appears to increase in line with the mean AR and LFC power. This might suggest that a reduced lag of accommodation could be linked to the higher resolution screens, which may be more beneficial to users than lower resolution screens.

It may be that a reduced lag of accommodation when observing higher resolution displays may be related to the improvements in readability and the trends of decreasing subjective fatigue seen in previous studies.^52^, ^54^ Given that the improvements in display technology have led to general increases in image resolution compared to the CRT models used in the past, they can be considered an improvement in dealing with DES. However, since DES continues to occur it may be only a small part of a much larger problem.^1^, ^2^, ^3^, ^4^, ^53^

It is important to consider the difference between myopes and emmetropes in this study. It is normal to have a small lag of accommodation. Patients tend to be symptomatic if the lag of accommodation exceeds 1.00 D or if there is a lead of accommodation.^31^ For emmetropes, the mean AR ranged from 2.40 D to 2.07 D for the 3.00 D accommodative demand which would constitute a normal lag of accommodation. However, in myopes the mean AR ranged from 3.18 D in the highest resolution (600 dpi) to 2.66 D in the lowest resolution (96 dpi). This may suggest that a higher resolution could cause a lead of accommodation in myopes. Given that leads in accommodation can be symptomatic, there is potential that image resolution may be a factor in causing symptoms in myopes during close work.^1^ Further research is required to determine the clinical significance of this finding.

## CONCLUSION

In conclusion, the mean accommodative response and the mean power of the LFC of AMFs are dependent upon on the type of display used. Higher resolution devices showed a reduced lag of accommodation to the accommodative demand; however this may cause a lead of accommodation in myopes for higher resolution display types. This may suggest that image resolution plays a role in symptoms of DES. It may be necessary to consider that non‐symptomatic image resolutions fall into a range occurring between excessive lags of accommodation with lower resolution and leads of accommodation for higher resolution displays.

## CONFLICTS OF INTEREST

The authors report no conflicts of interest and have no proprietary interest in any of the materials mentioned in this article.

## AUTHOR CONTRIBUTION

**Niall Joseph Hynes:** Conceptualization (equal); Data curation (equal); Formal analysis (equal); Investigation (equal); Methodology (equal); Writing – original draft (equal). **Matthew Cufflin:** Conceptualization (equal); Data curation (equal); Formal analysis (equal); Investigation (equal); Methodology (equal); Supervision (equal); Writing – original draft (equal). **Karen M Hampson:** Conceptualization (equal); Formal analysis (equal); Software (equal); Writing – original draft (equal). **Edward Mallen:** Conceptualization (equal); Methodology (equal); Resources (equal); Supervision (equal); Writing – original draft (equal).

## References

[1] Rosenfield M . Computer vision syndrome: a review of ocular causes and potential treatments. Ophthalmic Physiol Opt 2011;31:502–15. 21480937 10.1111/j.1475-1313.2011.00834.x

[2] Rosenfield M . Computer vision syndrome (a.k.a. digital eye strain). Optom Pract 2016;17:1–10.

[3] Coles‐Brennan C , Sulley A , Young G . Management of digital eye strain. Clin Exp Optom 2019;102:18–29. 29797453 10.1111/cxo.12798

[4] Sheppard AL , Wolffsohn JS . Digital eye strain: prevalence, measurement and amelioration. BMJ Open Ophthalmol 2018;3:e000146. 10.1136/bmjophth-2018-000146 PMC6020759 29963645

[5] Rosenfield M , Hue JE , Huang RR , Bababekova Y . The effects of induced oblique astigmatism on symptoms and reading performance while viewing a computer screen. Ophthalmic Physiol Opt 2012;32:142–8. 22150631 10.1111/j.1475-1313.2011.00887.x

[6] Rossignol AM , Morse EP , Summers VM , Pagnotto LD . Video display terminal use and reported health symptoms among Massachusetts clerical workers. J Occup Med 1987;29:112–8. 3819890

[7] Jaschinski W . Fixation disparity at different viewing distances and the preferred viewing distance in a laboratory near‐vision task. Ophthalmic Physiol Opt 1998;18:30–9. 9666908

[8] Jaschinski W , Konig M , Mekontso TM , Ohlendorf A , Welscher M . Computer vision syndrome in presbyopia and beginning presbyopia: effects of spectacle lens type. Clin Exp Optom 2015;98:228–33. 25963113 10.1111/cxo.12248

[9] The Vision Council . Digital eye strain. 2019; https://www.thevisioncouncil.org/content/digital‐eye‐strain Accessed 18 Jul 2019.

[10] Ichhpujani P , Singh RB , Foulsham W , Thakur S , Lamba AS . Visual implications of digital device usage in school children: a cross‐sectional study. BMC Ophthalmol 2019;19:76. 10.1186/s12886-019-1082-5 PMC6417240 30866885

[11] Jaiswal S , Asper L , Long J , et al. Ocular and visual discomfort associated with smartphones, tablets and computers: what we do and do not know. Clin Exp Optom 2019;102:463–77. 30663136 10.1111/cxo.12851

[12] Park M , Ahn YJ , Kim SJ , et al. Changes in the accommodative function of young adults in their twenties following smartphone use. J Korean Ophthalmic Opt Soc 2014;19:253–60.

[13] Iwasaki T , Kurimoto S . Objective evaluation of eye strain using measurements of accommodative oscillation. Ergonomics 1987;30:581–7. 3595555 10.1080/00140138708969747

[14] Day M , Seidel D , Gray LS , Strang NC . The effect of modulating ocular depth of focus upon accommodation microfluctuations in myopic and emmetropic subjects. Vision Res 2009;49:211–8. 18992269 10.1016/j.visres.2008.10.010

[15] Hynes NJ , Cufflin MP , Hampson KM , Mallen EAH . Cognitive demand and accommodative microfluctuations. Vision 2018;2:36. 10.3390/vision2030036 PMC6836075 31735899

[16] Wolffsohn JS , Gilmartin B , Mallen EA , Tsujimura S . Continuous recording of accommodation and pupil size using the Shin‐Nippon SRW‐5000 autorefractor. Ophthalmic Physiol Opt 2001;21:108–13. 11261344

[17] Ramamurthy D , Lin Chua SY , Saw SM . A review of environmental risk factors for myopia during early life, childhood and adolescence. Clin Exp Optom 2015;98:497–506. 26497977 10.1111/cxo.12346

[18] You QS , Wu LJ , Duan JL , et al. Factors associated with myopia in school children in China: the Beijing childhood eye study. PLoS One 2012;7:e52668. 10.1371/journal.pone.0052668 23300738 PMC3531363

[19] Lee Y‐Y , Lo C‐T , Sheu S‐J , Lin JL . What factors are associated with myopia in young adults? A survey study in Taiwan military conscripts factors associated with myopia in young adults. Invest Ophthalmol Vis Sci 2013;54:1026–33. 23322575 10.1167/iovs.12-10480

[20] Belete GT , Anbesse DH , Tsegaye AT , Hussen MS . Prevalence and associated factors of myopia among high school students in Gondar town, northwest Ethiopia, 2016. Clin Optom 2017;9:11–8. 10.2147/OPTO.S120485 PMC6095558 30214355

[21] Czepita M , Kuprjanowicz L , Safranow K , et al. The role of reading, writing, using a computer, or watching television in the development of myopia. Ophthalmol J 2016;1:53–7.

[22] Lam CS‐Y , Lam C‐H , Cheng SC‐K , Chan LY‐L . Prevalence of myopia among Hong Kong Chinese schoolchildren: changes over two decades. Ophthalmic Physiol Opt 2012;32:17–24. 22150587 10.1111/j.1475-1313.2011.00886.x

[23] Day M , Strang NC , Seidel D , Gray LS , Mallen EA . Refractive group differences in accommodation microfluctuations with changing accommodation stimulus. Ophthalmic Physiol Opt 2006;26:88–96. 16390487 10.1111/j.1475-1313.2005.00347.x

[24] Harb E , Thorn F , Troilo D . Characteristics of accommodative behavior during sustained reading in emmetropes and myopes. Vision Res 2006;46:2581–92. 16545421 10.1016/j.visres.2006.02.006 PMC1892179

[25] Charman WN , Heron G . Microfluctuations in accommodation: an update on their characteristics and possible role. Ophthalmic Physiol Opt 2015;35:476–99. 26303445 10.1111/opo.12234

[26] Siegenthaler E , Bochud Y , Bergamin P , Wurtz P . Reading on LCD vs e‐Ink displays: effects on fatigue and visual strain. Ophthalmic Physiol Opt 2012;32:367–74. 22762257 10.1111/j.1475-1313.2012.00928.x

[27] Kim H , Kim J . Reading from an LCD monitor versus paper: Teenagers’ reading performance. Int J Res Stud Educ Technol 2013;2:1–10.

[28] Abbott ML , Schmid KL , Strang NC . Differences in the accommodation stimulus response curves of adult myopes and emmetropes. Ophthalmic Physiol Opt 1998;18:13–20. 9666906

[29] Yeo AC , Kang KK , Tang W . Accommodative stimulus response curve of emmetropes and myopes. Ann Acad Med Singapore 2006;35:868–74. 17218998

[30] Day M , Strang NC , Seidel D , Gray LS . Effect of contact lenses on measurement of the accommodation microfluctuations. Ophthalmic Physiol Opt 2008;28:91–5. 18201340 10.1111/j.1475-1313.2007.00522.x

[31] Elliott DB . Clinical procedures in primary eye care, 4th edn. Oxford: Elsevier Saunders; 2014.

[32] Mallen EA , Wolffsohn JS , Gilmartin B , Tsujimura S . Clinical evaluation of the Shin‐Nippon SRW‐5000 autorefractor in adults. Ophthalmic Physiol Opt 2001;21:101–7. 11261343

[33] Wolffsohn JS , O'Donnell C , Charman WN , Gilmartin B . Simultaneous continuous recording of accommodation and pupil size using the modified Shin‐Nippon SRW‐5000 autorefractor. Ophthalmic Physiol Opt 2004;24:142–7. 15005679 10.1111/j.1475-1313.2004.00186.x

[34] Cufflin MP , Mallen EA . Dynamic accommodation responses following adaptation to defocus. Optom Vis Sci 2008;85:982–91. 18832977 10.1097/OPX.0b013e3181886fda

[35] Hampson KM , Mallen EA . Chaos in ocular aberration dynamics of the human eye. Biomed Opt Express 2012;3:863–77. 22567581 10.1364/BOE.3.000863 PMC3342193

[36] Gray LS , Winn B , Gilmartin B . Accommodative microfluctuations and pupil diameter. Vision Res 1993;33:2083–90. 8266650 10.1016/0042-6989(93)90007-j

[37] Gray LS , Gilmartin B , Winn B . Accommodation microfluctuations and pupil size during sustained viewing of visual display terminals. Ophthalmic Physiol Opt 2000;20:5–10. 10884926

[38] Hue JE , Rosenfield M , Saa G . Reading from electronic devices versus hardcopy text. Work 2014;47:303–7. 24284668 10.3233/WOR-131777

[39] Campbell FW , Robson JG , Westheimer G . Fluctuations of accommodation under steady viewing conditions. J Physiol 1959;145:579–94. 13642323 10.1113/jphysiol.1959.sp006164 PMC1356964

[40] Gray LS , Winn B , Gilmartin B . Effect of target luminance on microfluctuations of accommodation. Ophthalmic Physiol Opt 1993;13:258–65. 8265167 10.1111/j.1475-1313.1993.tb00468.x

[41] van der Heijde GL , Beers AP , Dubbelman M . Microfluctuations of steady‐state accommodation measured with ultrasonography. Ophthalmic Physiol Opt 1996;16:216–21. 8977885 10.1046/j.1475-1313.1996.95000518.x

[42] Denieul P . Effects of stimulus vergence on mean accommodation response, microfluctuations of accommodation and optical quality of the human eye. Vision Res 1982;22:561–9. 7112955 10.1016/0042-6989(82)90114-6

[43] Kotulak JC , Schor CM . Temporal variations in accommodation during steady‐state conditions. J Opt Soc Am A 1986;3:223–7. 3950795 10.1364/josaa.3.000223

[44] Jeng W‐D , Ouyang Y , Huang T‐W , et al. Research of accommodative microfluctuations caused by visual fatigue based on liquid crystal and laser displays. Appl Opt 2014;53:H76–84. 25322435 10.1364/AO.53.000H76

[45] Winn B , Gilmartin B , Mortimer LC , Edwards NR . The effect of mental effort on open‐ and closed‐loop accommodation. Ophthalmic Physiol Opt 1991;11:335–9. 1771070

[46] Day M , Gray LS , Seidel D , Strang NC . The relationship between object spatial profile and accommodation microfluctuations in emmetropes and myopes. J Vis 2009;9:1–13. 10.1167/9.10.5 19810786

[47] Langaas T , Riddell PM , Svarverud E , et al. Variability of the accommodation response in early onset myopia. Optom Vis Sci 2008;85:37–48. 18174839 10.1097/OPX.0b013e31815ed6e9

[48] Rosenfield M , Abraham‐Cohen JA . Blur sensitivity in myopes. Optom Vis Sci 1999;76:303–7. 10375246 10.1097/00006324-199905000-00018

[49] Nakatsuka C , Hasebe S , Nonaka F , Ohtsuki H . Accommodative lag under habitual seeing conditions: comparison between myopic and emmetropic children. Jpn J Ophthalmol 2005;49:189–94. 15944822 10.1007/s10384-004-0175-7

[50] Nakatsuka C , Hasebe S , Nonaka F , Ohtsuki H . Accommodative lag under habitual seeing conditions: comparison between adult myopes and emmetropes. Jpn J Ophthalmol 2003;47:291–8. 12782167 10.1016/s0021-5155(03)00013-3

[51] Moulakaki AI , Recchioni A , Aguila‐Carrasco AJ , Esteve‐Taboada JJ , Montes‐Mico R . Assessing the accommodation response after near visual tasks using different handheld electronic devices. Arq Bras Oftalmol 2017;80:9–13. 28380093 10.5935/0004-2749.20170004

[52] Ziefle M . Effects of display resolution on visual performance. Hum Factors 1998;40:554–68. 9974229 10.1518/001872098779649355

[53] Gowrisankaran S , Sheedy JE . Computer vision syndrome: A review. Work 2015;52:303–14. 26519133 10.3233/WOR-152162

[54] Miyao M , Hacisalihzade SS , Allen JS , Stark LW . Effects of VDT resolution on visual fatigue and readability: an eye movement approach. Ergonomics 1989;32:603–14. 2776740 10.1080/00140138908966135

